# Adoption, Fostering, and Parental Absence in Vanuatu

**DOI:** 10.1007/s12110-023-09456-0

**Published:** 2023-08-29

**Authors:** Eva Brandl, Emily H. Emmott, Ruth Mace

**Affiliations:** 1 Lise Meitner Research Group BirthRites, Department of Human Behavior, Ecology and Culture, Max Planck Institute for Evolutionary Anthropology, 04103 Leipzig, Germany; 2 Department of Anthropology, University College London, London, WC1E 6BT UK

**Keywords:** Alloparenting, Cooperative breeding, Fostering, Adoption, Kinship, Vanuatu

## Abstract

Alloparenting, wherein people provide care to children who are not their biological offspring, is a key aspect of human child-rearing. In the Pacific, many children are adopted or fostered by custodial alloparents even when both biological parents are still alive. From a behavioral ecology perspective, such behaviors are puzzling: why parent someone else’s child at your expense? Furthermore, little is known about how these arrangements are made in Pacific Islander societies today, who provides care, and what kinds of outcomes fostered children experience. A better understanding of these proximate factors may help reveal the ultimate drivers behind custodial alloparenting. Here, we report findings from a survey carried out with the caregivers of 282 children in rural areas of Vanuatu, an island nation in Melanesia. Most fostered and adopted children lived with relatives such as aunts, uncles, and grandparents (87.5%) rather than unrelated caregivers, with a strong preference for maternal kin. The most common reasons for these arrangements were that the parents had separated (16.7%), were engaging in labor migration (27.1%), or a combination of both (27.1%). Results for investment in children’s education and their educational outcomes were mixed, although children removed from crisis situations did more poorly than children removed for aspirational reasons. Our findings suggest that custodial alloparenting helps families adapt to socioeconomic transitions and changing marriage practices. Outcomes may depend on a range of factors, such as the reason children were transferred out of the natal home to begin with.

**Supplementary Information:**

The online version contains supplementary material available at 10.1007/s12110-023-09456-0.

In all human societies, child-rearing has a strong collaborative dimension in the sense that multiple allomothers (people who are not the biological mother) help with childcare (Hrdy, 2011; Sear, 2021). Like other forms of helping (Hamilton, 1963, 1964), alloparenting can evolve if alloparents receive indirect benefits through kin selection (increasing their inclusive fitness by caring for relatives) or direct benefits through reciprocity (for example, by receiving resources in return for caring for a child) (Emmott & Page, 2021). The costliest forms involve custodial alloparenting, where people raise another person’s child by fully substituting parenting. These include adoption (where people act as permanent custodial alloparents and assume primary responsibility for raising a child), fostering (a more temporary custodianship—here used to refer to informal foster arrangements common in the developing world rather than state-run foster care systems), and stepparenting (where people help raise a partner’s child from a previous relationship). In some African, Caribbean, and Pacific Island nations, many children live away from one or both biological parents for a substantial portion of their childhoods, even when both parents are still alive (Lloyd & Desai, 1992; Silk, 1980).

Why raise another person’s child? Providing this much care to children other than one’s own is puzzling from a behavioral ecology perspective. Sahlins (1976) argued that societies in which child transfers are common disprove kin selection theory because many children are not raised by their closest genetic relatives. Besides, Sahlins argued, kinship is a cultural, not a biological, phenomenon (Sahlins, 2012). Kin selection theory holds that seemingly altruistic behavior can evolve if it promotes the helper’s inclusive fitness (their own combined with that of their relatives). It therefore predicts that, all else being equal, custodial alloparents will preferentially raise children who are related to them (and may favor their own when allocating resources), not that people never raise another person’s child. While people in contemporary Western societies tend to foster and adopt children who are not related to them, Silk (1980) has shown in a classic paper that in Pacific Islander societies most foster and adoptive parents are closely related to their charges (see also Kay, 1963). In fact, extended kin (such as grandparents or aunts and uncles) are the most important custodial alloparents in many small-scale societies and much of the developing world, a practice known as “kinship fostering” (Hampshire et al., 2015; Hedges et al., 2019a; Isiugo-Abanihe, 1985; Rawson & Berggren, 1973; Scelza & Silk, 2014; Silk, 1987a, 1990). This suggests that custodial alloparenting may be explained as a form of kin altruism, whereby the caregiver benefits indirectly because they are related to the child.

Other drivers of cooperation, such as reciprocity and direct benefits, also play a role (Caldwell & Caldwell, 1987). In some historical settings, adoption helped perpetuate the caregiver’s lineage. Under Japanese rule, Taiwanese girls were often adopted by non-kin to facilitate a future marriage with the adopted parents’ biological son (Mattison et al., 2015). The adoptive parents thereby ensured the birth of grandchildren without the risks involved in pursuing a conventional marriage (Mattison et al., 2015). In other cases, the benefit to the caregivers is more economic in nature. For example, children are sometimes taken in to supplement the domestic labor force (known as “domestic fostering”; Isiugo-Abanihe, 1985). These children do farm work, help around the house, help look after younger children, and provide economic support (Alber, 2003; Hedges et al., 2019a; Sanford, 1975). In some Western countries, families used to employ other people’s children on their farms (Lischer, 2013). Caregivers may also receive resources from the biological parents (Isiugo-Abanihe, 1985; Kay, 1963).

In other cases, caregivers use fostering to increase their own prestige, gain political support, consolidate the interests of their lineage, or reinforce hierarchies within families (Bledsoe, 1990; Castle, 1995; Silk, 1987a, 1987b). “Alliance fostering” strengthens social ties between households, and people sometimes choose to take in an unrelated or distantly related child to cultivate a desired relationship (Alber, 2003; Isiugo-Abanihe, 1985). Relatives also exchange children to strengthen kin ties and widen their social network (Lloyd & Desai, 1992). In Oceania, adoption can build new relationships or strengthen existing kin ties, redistributing people depending on the requirements of different domestic and descent groups (Brady, 1976). For this reason, Ifalik people in the Caroline Islands are especially keen to adopt the children of high-ranking men (Betzig, 1988). Cultivating such ties may provide social, political, and economic advantages to the caregiver and their own offspring.

Kin altruism and reciprocity may therefore explain why people agree to take in another person’s child. But why would the biological parents move a child out of the natal home in the first place? Anthropologists have proposed that fostering is a form of dispersed cooperative breeding: it allows the biological mother to disinvest from some of her children and concentrate on others, with foster parents supporting the mother’s ongoing reproductive career (Scelza & Silk, 2014). Sometimes, this comes down to demographic factors: in high fertility environments, fostering helps parents with many children reduce the costs of child-rearing, which reallocates resources within the kin group (Caldwell & Caldwell, 1987; Isiugo-Abanihe, 1985). In such settings, children are more likely to be sent away if their biological parents have many children (Isiugo-Abanihe, 1985; Rawson & Berggren, 1973). For example, in Oceanic societies such as Chuuk, fostering and adoption have often served to reduce family sizes, transferring children from large families to those with few or no children (Goodenough, 1970; Silk, 1980).

Dispersed cooperative breeding and the associated disinvestment from active childcare (see Scelza & Silk, 2014) also emerge when resource acquisition and child-rearing make competing demands on a parent’s time. This occurs in countries undergoing rapid economic development, when opportunities for wage labor are concentrated in some regions. Sending children away enables the biological parents to migrate and pursue those opportunities (Isiugo-Abanihe, 1985); we might call this “opportunity fostering.” When mothers migrate to urban areas to search for employment, they can face strained resources and tenuous social networks in their new environment and thus decide to leave their children with rural kin (Isiugo-Abanihe, 1985; Lloyd & Desai, 1992). In urban areas of Tahiti, children are less of an economic asset to their parents than in rural areas, which is addressed by sending them away to live with older relatives (Kay, 1963). In those cases, parents “outsource” child-rearing in the short term to gain resources they want to invest back in their children in the long term.

Parents also disinvest from a child if they do not have the social support or resources they need to look after them or if the child’s presence compromises the parent’s reproductive career. This can occur when there is a crisis in the natal home (“crisis fostering”; Isiugo-Abanihe, 1985). For example, Inuit children were sometimes adopted out when family resources were strained (Silk, 1987a). Crisis fostering can also be a response to divorce or illegitimacy (Isiugo-Abanihe, 1985). In many small-scale societies and developing countries, children whose parents are unmarried or separated are more likely to be sent away (Cotton, 2021; Isiugo-Abanihe, 1985; Lloyd & Desai, 1992; Pennington, 1991; Rawson & Berggren, 1973; Silk, 1987a). Removing children from such situations may relieve resource pressures on the natal household or enable parents to pursue new partners and have more children after a failed relationship.

In summary, fostering and adoption often benefit parents and alloparents. The children may also benefit—for example, if they are removed from crisis situations that could compromise their welfare (such as income shocks, abuse, family disputes or previous incidents of child mortality in the natal home; Akresh, 2009; Isiugo-Abanihe, 1985; Syme & Hagen, 2023). In other cases, children are sent away to improve their social and economic capital. Children from lower social strata are sometimes placed with high-status households to promote their social mobility (Isiugo-Abanihe, 1985). Or children are sent to live with a person who provides them with religious, moral, or professional instruction or access to formal education (known as “apprentice fostering”; Isiugo-Abanihe, 1985). In West Africa, apprenticeship arrangements and residential formal education provide children with opportunities for educational and economic advancement (Goody, 1978). Similarly, some Chon Chuuk adolescents move in with extended kin to attend school in the United States (Syme & Hagen, 2023).

But children who live away from their biological parents can also pay a cost if they compete with other members of their new household or their caregivers are less invested in them than they are in their own children. In many settings, fostered children are at risk of abuse, domestic servitude, malnutrition, and bad educational outcomes (Bledsoe, 1990; Bledsoe et al., 1988; Hampshire et al., 2015; Madhavan & Townsend, 2007; Nelson, 2020; Rawson & Berggren, 1973; Scelza & Silk, 2014). Negative effects on mental and physical health may be long-term and persist into adulthood (Inoue et al., 2022; Yazawa et al., 2019, 2022). In Oceania, adoptees can also be disadvantaged in decisions about land inheritance (Silk, 1980). In the case of stepchildren, this has become known as the Cinderella Effect (Daly & Wilson, 1985, 1998). In many countries, stepchildren are more likely to experience a range of negative outcomes such as conflicts with caregivers, abuse, behavioral difficulties, low educational attainment and reproductive success, and even early death (Daly & Wilson, 1985, 1998; Emmott & Mace, 2014; Flinn, 1988; Snopkowski, 2016; Tooley et al., 2006; Zvoch, 1999). These effects cannot be reduced to other causes, such as poverty or family size (Daly & Wilson, 1998), and hold up in direct comparisons between stepchildren and their half-siblings (who have both biological parents in the home; Evenhouse & Reilly, 2004).

Other results contradict this picture. In some populations, stepparent effects are either absent or variable, possibly buffered by resource availability (Schacht et al., 2021; Snopkowski, 2016; Temrin et al., 2000; Willführ & Gagnon, 2013). Conflicting results have also been found for fostering and adoption. Living with close relatives, such as grandparents, can protect children from the negative outcomes normally associated with parental absence (Hedges et al., 2019a; Lawson et al., 2017). It may be that children are strategically placed with relatives who have the resources they need to look after them (Lawson et al., 2017). Circumstantial factors also matter. In Mali, children raised by a foster mother who requested them had better nutritional outcomes than children fostered out due to force of circumstance (Castle, 1995). Moreover, patterns of care are not always consistent with predictions from behavioral ecology because cultural processes can lead to the spread of social norms that do not improve fitness (Colleran, 2016). For example, patterns of adoption in Western societies are not consistent with kin altruism (Silk, 1990), and in some settings adoptees are better off than biological children. For example, Inuit parents did not always bias care toward their own children (Silk, 1987a). In Japanese-ruled Taiwan, adopted girls outlived girls who remained in the natal home, especially if they were *not* adopted to perpetuate the lineage (Mattison et al., 2018). Biological daughters may have been neglected because they had to compete with their brothers in a patriarchal society where parents strongly favored sons (Mattison et al., 2018). Finally, dual family membership may provide Chuuk adoptees with more social and material resources than are available to biological children (Goodenough, 1970).

Silk’s (1980) investigation of adoption in the Pacific played an important role in the development of biosocial research on this topic, but the ethnographic records the study was based may be dated now. In our study, we provide an update based on recent field data from Vanuatu, an island nation in the South Pacific. We answer the following questions: why do ni-Vanuatu people take in children who are not their own? Why are children sent away from the natal home? And are fostered children disadvantaged relative to children who live with both biological parents? A better understanding of these factors may help reveal the ultimate drivers behind custodial alloparenting. If kin altruism can explain why caregivers take in other people’s children, this type of care should be mostly provided by kin. If children are sent away from the natal home to reduce large family sizes, fostered and adopted children should have more biological siblings than non-fostered children and should transfer from larger households to smaller ones with fewer dependent children. Finally, if people conform to predictions from kin selection theory, they should provide more care to their own biological offspring than to other people’s children; we therefore expect higher levels of investment and better educational outcomes for children living with their biological parents than those living with other caregivers. We focused on education because it can improve offspring quality (enabling them to acquire resources in a market economy and making them more desirable on the marriage market). In societies undergoing market integration, economic payoffs and status competition in the labor market promote high parental investment in children’s embodied capital, including their education (Gibson & Lawson, 2011; Hedges et al., 2016; Kaplan, 1996; Shenk et al., 2016). This induces competition between siblings when parents preferentially provide opportunities to some children (Gibson & Lawson, 2011; Hedges et al., 2016, 2019b). Investment in education sometimes outpaces investment in child health once mortality has declined (Gibson & Lawson, 2011), indicating that this domain is relevant for assessing caregiver priorities.

## Methods

### Ethnographic Background

In 2019, the first author conducted fieldwork in rural areas on the north shore of Efate and on the northeast coast of Espiritu Santo in Vanuatu. People live in villages made up of interlinked extended families. Both locations practice matrilineal descent (clans known as *naflak* or *nakainanga* on Efate and moieties or “lines” known as *nalyö* on Santo) along with taboos which prohibit marriage within the same clan or line (Espirat et al., 1973; Guiart, 1964; Harrison, 1937:382), although on Efate, at least, paternal bloodlines are also recognized (Luders, 2001). Classic ethnographies describe residential units (*farea*) on Efate as patrilocal even though descent in the clan system is matrilineal (Espirat et al., 1973:338; Guiart, 1964); patrilocal residence is also common in many other ni-Vanuatu societies (Espirat et al., 1973:274). This appears to distinguish northern and central Vanuatu, and maybe Melanesia in general, from matrilineal descent systems in other parts of the world (70% of whom practice matrilocal residence: Surowiec et al., 2019; see also Fortunato, 2019). Most Melanesian societies with matrilineal descent systems have traditionally practiced “either variable or preferential virilocal postmarital residence” (Allen, 1981:17). Although patrilineal descent tends to be paired with patrilocal residence, in Melanesia matrilineal descent groups are often decoupled from territorial organization, meaning that their members are more widely dispersed (Allen, 1981; Guiart, 1964).

Families practice horticulture supplemented with fishing but also earn money through cash cropping and wage labor. Both areas are experiencing rapid social change due to rural–urban migration, seasonal labor, and the expansion of formal education (see also Miller, 1987, 1990; Petrou, 2017, 2018; Smith, 2018, 2019). Economic development has increased the importance of education in many Melanesian countries. Since education affords access to better-paid jobs, some view it as an opportunity to pursue upward mobility for themselves and their children (Rosi & Zimmer-Tamakoshi, 1993; Smith, 2018). Some also worry about the future viability of subsistence gardening, which could be compromised by land shortages and climate change (Smith, 2018). Money and success in the market economy have become important for prestige activities that improve men’s social status (Zimmer-Tamakoshi, 1993). Educated male wage-earners have become desirable on the marriage market, using money to attract women (Demian, 2017; Marksbury, 1993; Zimmer-Tamakoshi, 1993). They are also popular with potential in-laws because they can provide resources and support them in their old age (Servy, 2020). Men who are uneducated, unemployed, or on low incomes have poorer romantic prospects (Marksbury, 1993; Zimmer-Tamakoshi, 1993). Education is valued in women, too. Members of the upwardly mobile professional class prefer educated partners, and such men desire educated wives (Marksbury, 1993; Rosi & Zimmer-Tamakoshi, 1993). Educated women also command higher bride-prices (Filer, 1985; Henry & Vávrová, 2020; Jourdan & Labbé, 2020; Marksbury, 1993). Investment in education is therefore becoming an investment in offspring quality. But admission to secondary school is competitive as demand outstrips supply (Kalsuak, 2004), meaning that ni-Vanuatu children’s performance in primary school can have a considerable impact on their future. At the same time, parental investment in education (through paying school fees and monitoring homework and attendance) differs widely between households (Kalsuak, 2004; Tari, 2004; Worwor, 2004). As a result, some children will be in a better position to take advantage of these opportunities than their peers.

Adoption and fostering occur in both field sites. Adoptions involve a formal transfer of children from the biological to the adoptive parents and a long-term change in the identity of the person with the primary responsibility for and authority over a child. On Efate, this is accompanied by a small *kastom* ceremony which may feature speeches, feasting, and the display of clan symbolism, attended by the wider family. Traditionally, the biological father was compensated with pigs (Guiart, 1964). In contrast, fostering is informal and without ceremony, often temporary, and not accompanied by a long-term change in status.

Early ethnographers of Vanuatu note that concerns related to kinship often shaped the circulation of children. Adoption followed from the logic of a kinship system in which children belong to the kin group as a whole; for example, people often used adoption or foster arrangements to restore “balance” between different lineages or moieties (Layard, 1942:187). On Vao, adoptions were usually restricted to the offspring of a classificatory brother in a man’s own patrilocal group (Layard, 1942:187). In an Ambrymese migrant community on Efate, most adoptive fathers were already classificatory fathers of their adopted children (Tonkinson, 1976). Some transfers were connected to sister exchange: if a father did not have enough daughters to exchange for wives for his sons, he could claim a brother’s daughter on the grounds that he had contributed to his brother’s bride-price (Tonkinson, 1976). Others also laid the groundwork for a future marriage. On Vanua Lava, people sometimes took in a girl to raise as a future bride for their son, although this is no longer practiced (Hess, 2009:83). Although adoptions in northern and central Vanuatu usually occurred within the same descent group (Allen, 1981), adults sometimes used adoptions to change their group affiliation and circumvent marriage taboos (Efate: Guiart, 1964; Mota: Kolshus, 2008).

On Vao, the family’s offspring were likened to yams “since, like this staple article of food on which the life of the community primarily depends, it must be tended with great care and labor but amply repays the energy expended on it” (Layard, 1942:181). These acts of nurture build social ties. Contemporary ethnographers write that parenthood is accumulated through a series of “life-sustaining contributions to the child” (Larcom, 2000:88). Adults who provide “food and other gifts to a child,” or who provision the mother during her pregnancy, “earn . . . a share of the child and can to some extent claim it as [their] own” (Larcom, 2000:94, see also p. 95). Similarly, children who grow up together on Vanua Lava establish “relatedness through being fed by the same parents and ‘eating out of one basket’” (Hess, 2009:84), and “Mewun see strong group ties as products of nurturing and feeding processes” (Larcom, 2000:86). This sentiment is evident in alliance fostering. Mewun men attempt to cast a wide net of personal ties linking them with other members of their place; this includes ties established through fostering and adoption (Larcom, 2000:102). Big men use these ties to build up a large network of supporters (Larcom, 2000:102). Similarly, Paama islanders often transfer a child to relatives in Port Vila, the national capital (Lind, 2014). This strengthens connections between families, which gives rural people access to opportunities in the capital and paves the way for future marriages (Lind, 2014).

Adoption can also resolve childlessness and gender imbalances among offspring (Kolshus, 2008). On Vanua Lava, people who only have daughters may adopt a boy who will support them in their old age and inherit their land in return (Hess, 2009:74). However, these decisions are not taken lightly, and child circulation can be contentious. Among the Big Nambas of Malakula, women are under intense pressure to produce a child before adoption is considered (Colleran, 2022). Big Nambas families also refrain from adopting outsiders, which may be due to succession rules and fears over losing access to land (Colleran, 2022). Inheritance is also relevant on Mota, where adopted people often belong to multiple kin groups (Kolshus, 2008). Ambiguous affiliations have become a bone of contention in land disputes, with some families concentrating decision-making powers in men who were never adopted out (and therefore are not suspected of divided loyalties; Kolshus, 2008). Finally, fostering may contribute to malnutrition when breastfeeding infants are separated from their mothers and weaned abruptly by extended kin (motivated by a belief that mothers should stop breastfeeding if they become pregnant again; Wentworth, 2017). Although many mothers embrace fostering, senior kin sometimes make these decisions against the mother’s wishes (Wentworth, 2017).

Recently, ethnographers have noted a decline in arranged marriages and a rise in premarital relationships, marital instability, and single motherhood (Servy, 2020). In South Efate, elderly people tend to associate this phenomenon with a breakdown of traditional authority and a decline of reciprocal relationships between the generations (Widmer, 2013). Qualitative research indicates that unwed mothers tend to live with their parents or extended kin, on whom they depend for financial support (Widmer, 2013). Although grandparents tend to accept “illegitimate” children once they are born, unwed mothers can face social stigma, and future support from the child’s father is uncertain (Widmer, 2013). Maybe for that reason, “fatherless” children on Mota often end up being adopted (Kolshus, 2008).

### Survey

The first author consulted class lists of children enrolled in kindergarten and primary school and then visited the households where the children were living at the time to conduct structured interviews with their caregivers. On each island, the first author was accompanied by a local research assistant who located the relevant households and clarified the meanings of our questions if necessary. We asked respondents to name all the residents in the home, specify their genealogical relationship with them, and describe the educational attainment of each adult in the home. If a respondent expressed puzzlement at the question “Who lives with you?” we explained that we were interested in who was sleeping in their house on a regular basis during that time period. We then asked the guardian how many children they had and to name all their children by order of birth. With each child, we enquired whether they were the respondent’s biological (*stret*) child and whether they were living with the respondent or with someone else. If a child was the respondent’s biological child, we also enquired about the identity of the other biological parent. If a child had no biological parents in the home, we asked about the relationship status of the biological parents, the reason for transferring the child out of the natal home, whether the child had any biological siblings, and if so, how many. We also enquired directly whether the caregiver had adopted (*adoptem*) the child (this reflects the respondent’s assessment of the relationship and includes both *kastom* and statutory adoptions). If a child only had one biological parent in the home, we asked about the reason for the other parent’s absence. At the end, we reviewed the information provided by the respondent and asked whether they had any more children or whether any more coresidents were staying with them to ensure that no one had been left out. We also enquired about other topics, such as people’s source of income, which are not presented here (see Brandl, 2021).

Some residents were dividing their time between multiple households. These cases were resolved by asking participants in which household the relevant person spent most of their time and where they slept most nights. Adults who were only away for a short period of time were still counted as residents. Adolescents attending boarding school were also counted if they spent their term holidays at home. We set out to interview one respondent per household, but most of the time the other residents were present and gave their information themselves. All interviews were conducted in Bislama (pidgin English), the national lingua franca. Interviewees provided verbal consent ahead of questioning. The study was approved by the Vanuatu Cultural Centre, Port Vila; the Department of Anthropology, University College London (Approval Code: ANTHPGR_2018_005); and the central Research Ethics Committee, University College London (Project ID: 12,951/001).

### School Records and Interviews

We assessed children’s educational outcomes using school records from one primary school on Efate and one on Santo. Teachers provided exam scores for the final term of the 2019 school year (September–December). This is the combined score from all subjects (0–100%), which determines progression to the next year. Teachers also provided attendance records with information on primary schoolers’ truancy, recorded as the number of half-days absent in the same term. To assess caregivers’ investment in children’s education, the first author interviewed teachers whose classes had participated in a home reading project. For this project, children were handed reading assignments to take home, read aloud with their caregivers, and return once completed, after which they were given the next assignment. Caregivers had to sign a form to confirm that their child had read the book with them, which required them to play an active role in the child’s education (rather than leaving it to the teachers). Both schools communicate to the caregivers that home reading improves literacy (as shown in education research; Girard et al., 2021; Kim & Riley, 2021; Sénéchal & LeFevre, 2002). During the interview, teachers coded all their students’ reading participation rates on a 5-point scale (0–4: 0 = Never; 1 = Rarely; 2 = Sometimes; 3 = Often; and 4 = Always). Teachers provided verbal consent ahead of questioning and teachers and principals gave permission to view the relevant records.

### Analysis

We calculated descriptive statistics for children’s residence arrangements and the reasons for parental absence. We ran Kruskal–Wallis tests to assess whether fostered and adopted children had more biological siblings than non-fostered children; effect sizes (η^2^) were calculated with the *rstatix* package (Kassambara, 2021) in R (R Core Team, 2018, v. 4.1.1). We also ran Wilcoxon signed-rank tests to assess whether the number of biological siblings that fostered and adopted children had in the natal home was greater than the number of minors with whom they were sharing their current home—in other words, whether they had transferred from households with more dependent children to homes with fewer. We then examined educational attainment and investment. We ran Spearman correlations to assess whether children’s examination scores, absences, and home reading participation were correlated with each other. We also ran χ^2^ tests to examine whether parental absence was associated with caregiver and household education (and thus confounded); effect sizes (Cramér’s *V*) were calculated with *rcompanion* (Mangiafico, 2021). We then used multilevel regression models to examine whether coresidence with the biological parents was predictive of investment in children’s education and their educational outcomes while controlling for guardians’ level of education. We used *ordinal* (Christensen, 2019) to run ordinal logistic regression models for children’s reading return rates and absences (binned into 0–5, 6–10, 11–20, and > 20 absences). We used *glmmTMB* (Brooks et al., 2017) to run beta regression models for children’s exam scores, represented as ratios (*x*/100). Predictors were the highest educational attainment of any adult in the household, the education of the primary female caregiver (the biological, adoptive, foster, or stepmother, depending on circumstances), and the number of biological parents living in the home (2, 1, or 0). We also included random effects for Household ID and Caregiver ID since some of the children were sharing a home. We used ANOVA model comparisons to compare the results to a null model that only included random effects. We also performed post-hoc analyses to assess how sensitive our results were to our initial model specifications (including caregiver but not household education as a predictor and re-running truancy analyses with Poisson regressions and unbinned outcome data). Complete household interview data are available for *N* = 282 children (aged 3–11 years old) living in 213 households. Exam scores and absences are available for *n* = 176 and home reading return rates are available for *n* = 91 primary schoolers. Graphics were made with *ggplot2* (Wickham, 2016) and *scales* (Wickham & Seidel, 2020). Unless stated otherwise, fostered and adopted children were grouped together in our analyses due to the small number of adoptees in our sample.

## Results

### Full Sample

Although the ethnographic record states that Efate, and Vanuatu more broadly, are patrilocal (Allen, 1981; Espirat et al., 1973:274, 338), in our sample this only applied to children living with both biological parents, among whom most of those who also shared their home with extended kin lived with paternal relatives (59.3%). But across the whole sample, most children sharing their home with extended kin lived with maternal relatives (57.1%). Most children who lived with extended kin lived with their aunts and uncles (46.9%), cousins (38.9%), and/or grandparents (58.6%). Living with more distant kin (such as their parents’ cousins and other people related to the child through their grandparents’ siblings) was less common (Table 1). Other kinds of arrangements (such as households that included unrelated individuals and blended families with purely affinal connections between the residents) were also rare (6.4% and 3.2%, respectively). In a few cases (1.4%), the degree of relatedness between the residents was unclear. This was the case for one household on Efate and one on Santo. On Efate, a child lived with the adoptive parents of their biological mother, but the precise genealogical connection between the child and head of household could not be clarified. On Santo, a nuclear family had adopted a child whose biological mother had herself been adopted by the child’s adoptive mother’s aunt some decades prior, but the connection that motivated that first adoption could not be identified.

**Table 1:** Residence arrangements (reported as % of children) for the full sample (*n* = 282), for children living with both biological parents (*n* = 190), children living with one biological parent (*n* = 44), and children living with neither biological parent (*n* = 48, fostered: *n* = 39, adopted: *n* = 9). Households are grouped into the following categories: Single Parent (one parent and their children); Nuclear Family (a couple and their children); Extended Family (households that include extended kin); Expanded Family (nuclear or extended family households that include an unrelated resident or a resident whose kin connection was too distant to reconstruct); Blended Family (children living with a stepparent and/or the stepparent’s extended kin, children living with affinal kin, and children living with a biological parent and a stepparent and stepsiblings); and Unclear (households where the relatedness between the residents was unclear). Figures for “Extended Kin: Line” and “Extended Kin: Type” only apply to children who share a home with extended kin, not the whole sample

		Parents Present			Neither Parent Present	
	Full Sample (*N* = 282)	Both (*n* = 190)	One (*n* = 44)	None (*n* = 48)	Fostered (*n* = 39)	Adopted (*n* = 9)
		67.4	15.6	17.0	81.3	18.8
Household Type						
Extended family	52.8	43.7	61.4	81.3	87.2	55.6
Nuclear family	34.4	51.1	—	—	—	—
Single parent	1.8	—	11.4	—	—	—
Expanded family	6.4	3.2	15.9	10.4	7.7	22.2
Blended family	3.2	1.1	11.4	4.2	2.6	11.1
Unclear relatedness	1.4	1.1	—	4.2	2.6	11.1
Primary Caregiver						
Biological parent	83.0	100.0	100.0	—	—	—
Other relative	14.9	—	—	87.5	94.9	55.6
Unrelated person	1.4	—	—	8.3	2.6	33.3
Unclear relatedness	0.7	—	—	4.2	2.6	11.1
One Parent: Caregivers						
Mother			90.9			
Stepparent present			11.4			
Extended Kin: Line						
Maternal	57.1	33.7	82.4	83.7	89.5	40.0
Paternal	36.8	59.3	11.8	11.6	7.9	40.0
Both maternal and paternal	1.8	2.3	—	2.3	—	20.0
Older sibling’s children	4.3	4.7	5.9	2.3	2.6	—
Extended Kin: Type						
Aunts and uncles	46.9	29.1	64.7	69.0	67.6	80.0
Cousins	38.9	37.2	41.2	40.5	37.8	60.0
Grandparents	58.6	48.8	82.4	59.5	62.2	40.0
Great-grandparents	5.6	1.2	5.9	14.3	16.2	—
Grandparent’s sibling or their relatives	16.0	18.6	11.8	14.3	13.5	20.0
Great-grandparent’s sibling	0.6	—	2.9	—	—	—
Cousin’s children	2.5	2.3	—	4.8	2.7	20.0
Older sibling’s children	6.2	8.1	5.9	2.4	2.7	—

Figures for older sibling’s children are not always consistent between “Extended Kin: Line” and “Extended Kin: Type” because categories in “Line” are mutually exclusive (e.g., children who lived with an older sibling’s children but also with maternal kin were assigned to “Maternal”) whereas categories in “Type” are not

### Fostering, Adoption, and Single Parenthood

Two out of three children lived with both biological parents (67.4%), and one out of six lived with one (15.6%) or neither (17.0%) biological parent. Interlocutors told us that people usually foster or adopt relatives, framing child circulation in terms of kin solidarity and an obligation to look after relatives. Our demographic data also show that children living with neither biological parent overwhelmingly lived with relatives (87.5%). Among those living with extended kin, most lived with maternal relatives (83.7%), usually with aunts and uncles (69.0%), first cousins (40.5%), and/or grandparents (59.5%). Living with more distant relatives or unrelated caregivers (such as friends) was not common (see Table 1).

**Fig. 1:**
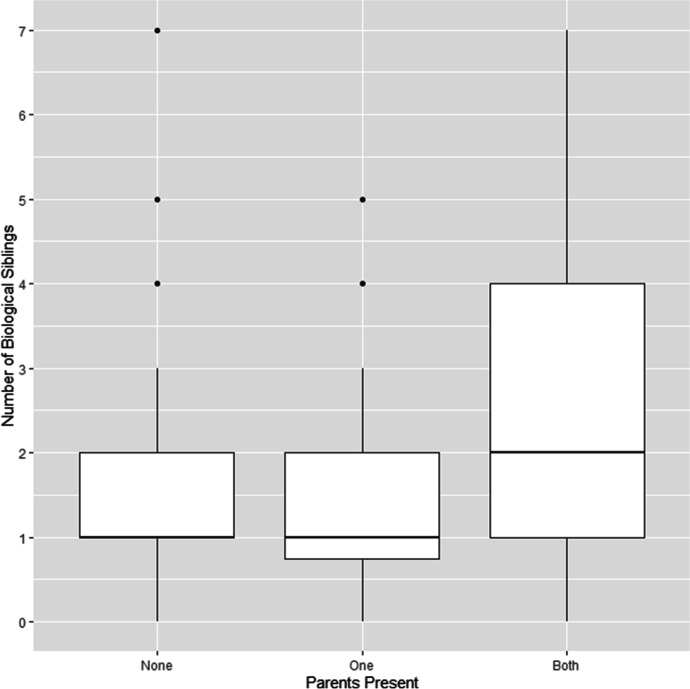
Comparison of the number of biological siblings for children living with both biological parents, children living with one parent, and children living with neither parent (all children with known number of biological siblings, *n* = 280; the number of biological siblings was not known for two fostered and adopted children)

**Fig. 2:**
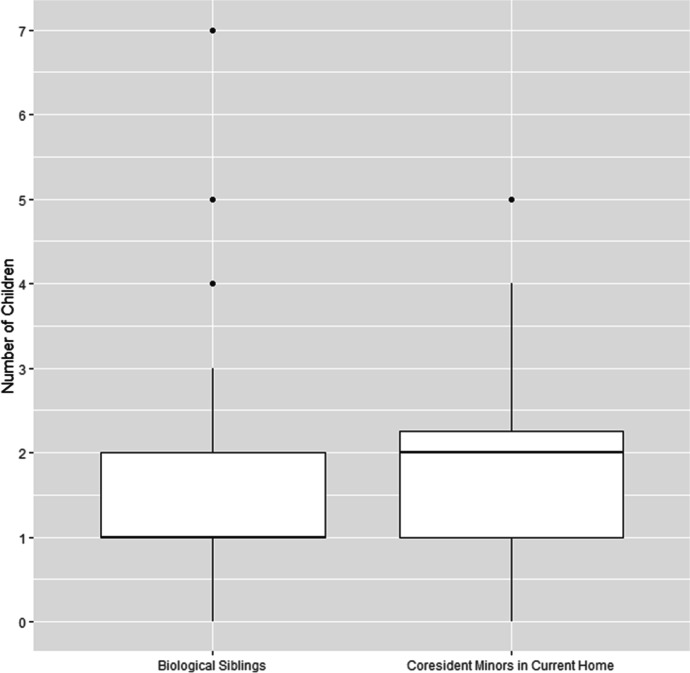
Comparison between fostered and adopted children’s number of biological siblings and the number of minors with whom fostered and adopted children were sharing their current home (fostered and adopted children with known number of biological siblings, *n* = 46; the number of biological siblings was not known for two fostered and adopted children)

Most children who were living away from both parents were fostered informally (81.3%), with their caregivers stating that they were looking after the child but had not adopted them (see Table 1). Against expectations, fostered and adopted children did not have more biological siblings than other children—in fact, it was the other way around: children living with both parents had more siblings on average (mean = 2.61, SD = 1.55) than fostered and adopted children (mean = 1.74, SD = 1.56; η^2^ = 0.12, *p* < 0.001; see Table 2 and Fig. 1). Moreover, for fostered and adopted children there was no significant difference between the number of biological siblings and the number of minors with whom they were sharing their current home (mean = 1.85, SD = 1.47; *p* = 0.56; see Table 2 and Fig. 2). Interviewees did not mention concerns about family size either. Instead, the most common reasons given for living with neither parent were: labor migration (27.1%), separation of the biological parents (16.7%), or a combination of both (27.1%; see Table 3). For rural families, seasonal agricultural labor in Australia and New Zealand is increasingly becoming a source of income; others migrate to urban areas within Vanuatu to find employment in the expanding commercial economy, such as the tourism sector. Both require extended periods of absence from the village. More rarely, children had been transferred due to extreme circumstances such as the evacuation of their home island after a natural disaster or abuse in the natal home (domestic violence directed at the child or their biological mother; see Table 3).

**Table 2:** Comparison of the number of biological siblings for children living with both biological parents, children living with one parent, and children living with neither parent (all children with known number of biological siblings: *n* = 280, Kruskal–Wallis test). And comparison between fostered and adopted children’s number of biological siblings and the number of minors with whom fostered and adopted children were sharing their current home (fostered and adopted children with known number of biological siblings: *n* = 46, Wilcoxon signed-rank test; the number of biological siblings was not known for two fostered and adopted children)

	Parents Present: Mean (SD)							
	Both	One	None	*V*	χ^2^	η^2^	df	*p*
Biological siblings	2.61 (1.55)	1.34 (1.26)	1.74 (1.56)	—	33.98	.12	2	< .001
Coresident minors	2.43 (1.39)	2.50 (2.20)	1.85 (1.47)	263	—	—	—	.56

**Table 3:** Reasons for transfer out of the natal home for children living away from both biological parents (*n* = 48 of 282; fostered: *n* = 39, adopted: *n* = 9) and reasons for the other parent’s absence for children living with one biological parent (*n* = 44 of 282) reported as % of children

	Parents Present		Neither Parent Present	
Reason for Parental Absence	One	None	Fostered	Adopted
Labor migration	13.6	27.1	33.3	—
Separation of parents	70.5	16.7	10.3	44.4
Both migration and separation	—	27.1	28.2	22.2
Split household	4.5	—	—	—
Death of a parent	11.4	—	—	—
Evacuation after natural disaster	—	2.1	2.6	—
Rejection by biological parents	—	2.1	—	11.1
Abuse in the natal home	—	4.2	2.6	11.1
To attend school	—	4.2	5.1	—
No reason provided	—	16.7	17.9	11.1

Disaggregated analyses indicate that crisis situations related to social dynamics in the natal home (separation, abuse, and rejection by the biological parents) were more common among adoptees (66.6% combined) than among foster children (12.9% combined; see Table 3). Conversely, purely aspirational reasons (education and labor migration) were more common among fostered children (38.4% combined) than adoptees (none; see Table 3). Adoptees lived with unrelated caregivers (33.3%) at higher rates than fostered children (2.6%; see Table 1). For those living with extended kin, fostered children lived with maternal kin (89.5%) more frequently compared with adoptees (40.0%).

Some children lived with one biological parent. Most of them lived with their mothers (90.9%), did not have a stepparent (only 11.4% did), and resided in extended family households (61.4%). Only few lived in more unusual arrangements, such as single-parent homes (11.4%). Those living with extended kin overwhelmingly lived with maternal relatives (82.4%), usually their aunts and uncles (64.7%), cousins (41.2%), and/or grandparents (82.4%). Only few shared a home with more distant kin (Table 1). The most common reason for living with just one parent was that the parents had separated (70.5%; see Table 3). Less commonly, this was due to the death of a parent (11.4%); labor migration (13.6%), wherein the other parent lived and worked in town or overseas; or the formation of a split household in which the parents live apart but are not formally separated (4.5%). Accordingly, in an environment where love matches, premarital relationships, and relationship breakdown have increased at the expense of arranged marriages, single mothers rely on coresident kin for support.

### Schooling

Key informants told us that the adoptive parents become mother and father to the adopted child, who is entitled to family property as a full member of the adoptive household. One interlocutor noted that she “had lived with her [foster caregiver] for many years, and still call[s] her mother” as a sign of closeness and appreciation. Another noted that “we opened up our home, and many blessings came back to us,” suggesting that the generosity shown through fostering is looked on kindly by God. However, child transfers are not viewed as unambiguously positive. On Santo, a teacher reported that children living apart from their mothers (adoptees, stepchildren, and those living with their grandparents) often perform worse and are disobedient. This teacher was native to the area and embedded in her students’ kin networks so she was familiar with their family backgrounds and conveying her personal experience in the classroom. Others felt that grandparents keep children fed and clothed but do not provide much support with schoolwork since some elderly people only have limited education themselves. On Efate, some residents felt that it is “not good when children do not live with their mothers” and that illegitimate children are “first in line for abuse.” In both locations, we encountered fostered and adopted children who experience conflicts with their caregivers and struggle in school.

On average, children scored around 73 out of 100 in their exams (mean = 72.76, SD = 18.59) and were absent for 6 half-days (mean = 5.55, SD = 6.26) throughout term 3 (Table 4). Most children returned their reading assignments often (33.0%) or always (29.7%), with a smaller number of children returning them never (13.2%), rarely (7.7%), or sometimes (16.5%). Children with more absences generally scored lower in their exams, although the correlation was not significant (*r* =  − 0.14, *p* = 0.07; see Table 5. For descriptive statistics, see ESM). Conversely, children with higher reading participation rates (i.e., higher levels of direct investment from guardians) scored significantly higher in their exams, showing a much stronger correlation (*r* = 0.49, *p* < 0.001). Furthermore, children who were absent more often had significantly lower reading participation rates (*r* =  − 0.36, *p* < 0.001). Truancy and home reading participation are inherently linked (children who are absent much of the time cannot pick up or return their assignments), although caregivers who are less invested in children’s academic progress are probably also less inclined to enforce attendance. In line with informants’ statements, fostered and adopted children were more likely to live with a female caregiver who only had a primary-level education (52.1%) than children living with both biological parents (36.3%; see Table 4). Conversely, the latter were more likely to live with a highly educated female caregiver (21.6%) than fostered and adopted children (4.2%). Differences in the caregiver’s level of education were significant (*V* = 0.13, df = 4, *p* = 0.04), but no differences were found at the household level (Table 5).

**Table 4:** Exam scores, absences (mean, SD), and reading participation (reported as % of children) for children living with both biological parents, children living with one parent, and children living with neither parent (children with known exam scores and absences: *n* = 176; children with known reading participation: *n* = 91). And caregiver- and household-level education (Primary or Lower = up to 6 years of education; Lower Secondary = 7–10 years of education; Upper Secondary or Higher = 11 years of education or more) for children living with both biological parents, children living with one parent, and children living with neither parent (whole sample, reported as % of children)

		Parents Present		
	Full Sample	Both	One	None
Exams: Mean (SD)	72.76 (18.59)	74.28 (17.40)	71.91 (19.32)	67.91 (21.70)
Truancy: Mean (SD)	5.55 (6.26)	5.58 (6.27)	5.14 (6.39)	5.74 (6.28)
	% of Children			
Readings				
Never	13.2	10.2	8.3	25.0
Rarely	7.7	5.1	8.3	15.0
Sometimes	16.5	20.3	25.0	0.0
Often	33.0	25.4	33.3	55.0
Always	29.7	39.0	25.0	5.0
Household Education				
Primary or Lower	17.0	16.3	15.9	20.8
Lower Secondary	37.9	37.9	38.6	37.5
Upper Secondary or Higher	45.0	45.8	45.5	41.7
Caregiver Education				
Primary or Lower	40.8	36.3	47.7	52.1
Lower Secondary	41.5	42.1	36.4	43.8
Upper Secondary or Higher	17.7	21.6	15.9	4.2

**Table 5:** Relation between home reading, truancy, and exam performance (Spearman correlations, children with known exam scores and absences: *n* = 176; children with known reading participation: *n* = 91). And comparison of caregiver- and household-level education for children living with both biological parents, children living with one parent, and children living with neither parent (χ^2^, whole sample)

Predictor	Outcome	*r*	χ^2^	*V*	df	*p*
Truancy	Exams	− .14	—	—	—	.07
Readings	Exams	.49	—	—	—	< .001
Truancy	Readings	− .36	—	—	—	< .001
Parental Absence	Household Education	—	0.65	.03	4	.96
Parental Absence	Caregiver Education	—	9.96	.13	4	.04

**Fig. 3:**
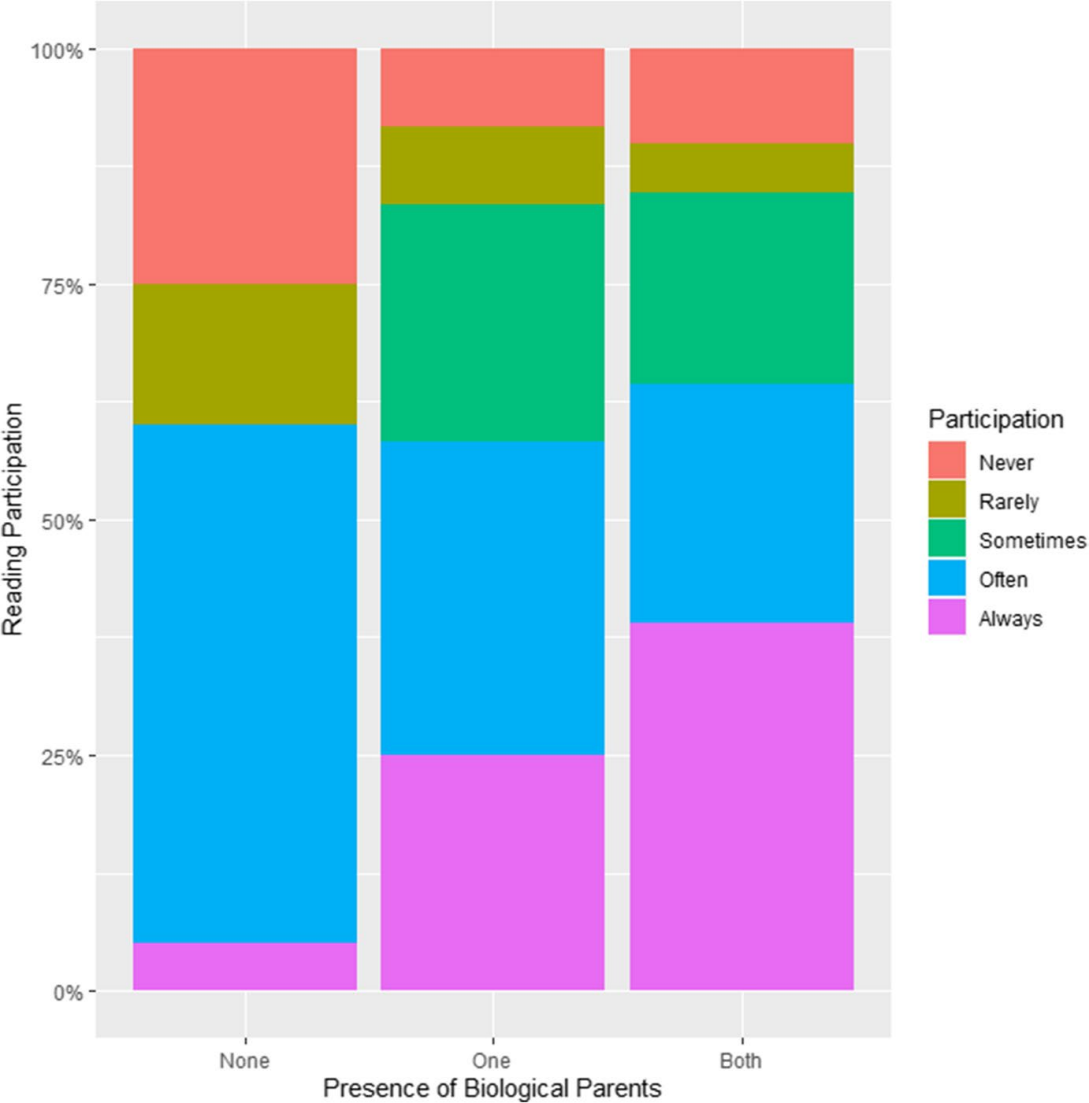
Comparison of home reading participation for children living with both biological parents, children living with one parent, and children living with neither parent (children with known reading participation, *n* = 91)

A full model predicting children’s reading participation rates from household and caregiver education and parental absence (the number of biological parents in the home) did not improve over the baseline (*p* = 0.10; see Table 6). A model including parental absence and caregiver but not household education did (*p* = 0.04), but the best-performing model only included parental absence (*p* = 0.02). Many fostered and adopted children never or rarely (40.0% combined) returned their readings, but this was much less common among children living with their biological parents (one parent: 16.6% combined; both parents: 15.3% combined; see Table 4 and Fig. 3). Very few fostered and adopted children always returned their readings (5.0%), but this was much more common among children who lived with one (25.0%) or both (39.0%) biological parents. This suggests that children living with their biological parents receive more investment in their education. Children whose primary female caregiver only had a primary-level education never or rarely took part in reading (29.7% combined) at higher rates than children whose caregiver had been educated to upper secondary level (10.0% combined; see ESM). Conversely, children whose caregiver had a high level of education often or always returned their readings (80.0% combined) at higher rates than children whose caregiver only had a primary-level education (54.0%, see ESM). However, the combined proportion of children who never or rarely took part was similar for different levels of household education, and models that only included education predictors did not improve over the baseline (*p* = 0.12 and 0.30; see Table 6).

**Table 6:** Model comparison: home reading participation, exam scores, and truancy predicted by caregiver- and household-level education and the number of biological parents in the home (children with known exam scores and absences, *n* = 176; children with known reading participation, *n* = 91)

Models	df	AIC	Log-Like.	LR	χ^2^	*p*
Reading						
Baseline	—	287.88	− 139.94	—	—	—
Caregiver (C) education	2	287.57	− 137.78	4.31	—	.12
Caregiver and household (H) education	4	290.98	− 137.49	4.90	—	.30
Parental absence	1	283.97	− 136.99	5.91	—	.02
Parental absence and C education	3	285.52	− 135.76	8.36	—	.04
Parental absence, C and H education	5	288.69	− 135.35	9.19	—	.10
Truancy						
Baseline	—	316.65	− 154.33	—	—	—
Caregiver education	2	319.51	− 153.75	1.15	—	.56
Caregiver and household education	4	321.79	− 152.89	2.87	—	.58
Parental absence	1	317.97	− 153.99	0.68	—	.41
Parental absence and C education	3	321.01	− 153.50	1.65	—	.65
Parental absence, C and H education	5	323.45	− 152.73	3.20	—	.67
Exams						
Baseline	—	− 137.90	72.95	—	—	—
Caregiver education	2	− 141.23	76.61	—	7.33	.03
Caregiver and household education	4	− 138.82	77.41	—	8.92	.06
Parental absence	1	− 139.12	74.56	—	3.22	.07
Parental absence and C education	3	− 140.99	77.50	—	9.09	.03
Parental absence, C and H education	5	− 138.58	78.29	—	10.68	.06

**Fig. 4:**
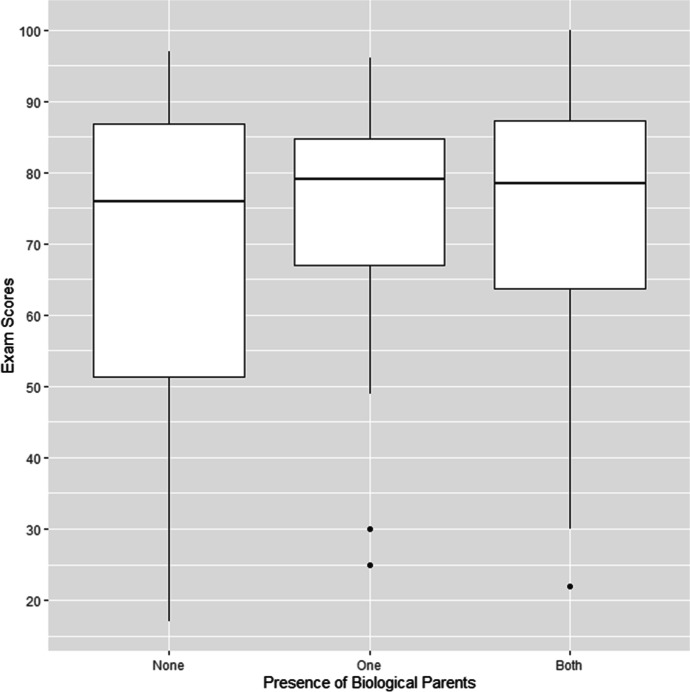
Comparison of exam scores for children living with both biological parents, children living with one parent, and children living with neither parent (children with known exam scores, *n* = 176)

**Fig. 5:**
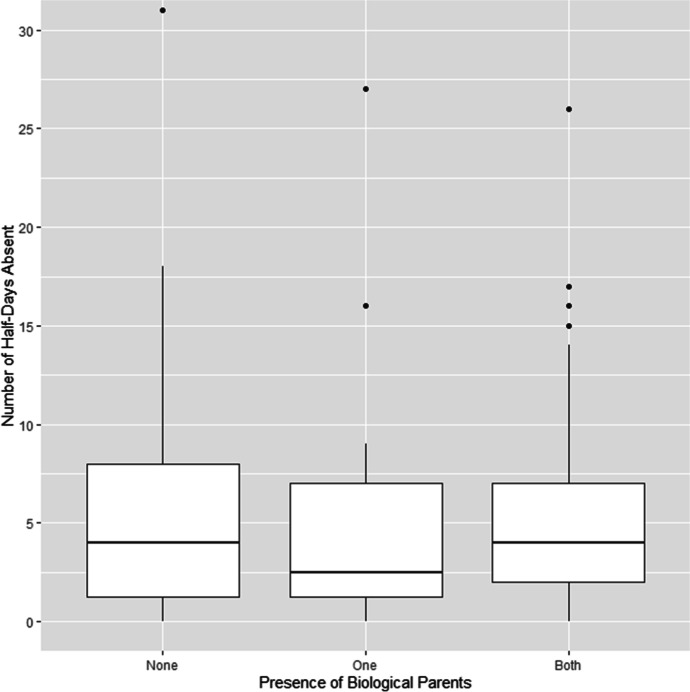
Comparison of truancy for children living with both biological parents, children living with one parent, and children living with neither parent (children with known absences, *n* = 176)

Exam scores of fostered and adopted children (mean = 67.91, SD = 21.70) and children who were living with just one parent (mean = 71.91, SD = 19.32) were slightly lower than those of children living with both parents (mean = 74.28, SD = 17.40; see Table 4 and Fig. 4). Although results were similar for different levels of household education, children whose primary female caregiver was educated to a high level scored higher in their exams (mean = 77.54, SD = 17.79) than children whose caregiver only had a primary-level education (mean = 68.63, SD = 20.26; see ESM). Models that included caregiver but not household education (with or without parental absence) significantly improved model fit compared with the baseline (*p* = 0.03; see Table 6), but the best-performing model only included caregiver education without parental absence. None of the other models improved fit (*p* = 0.06 and 0.07, respectively). Children with caregivers educated at the primary level were absent more often (mean = 6.09, SD = 8.07) than children with highly educated caregivers (mean = 4.27, SD = 3.49; see ESM), but absences were similar for children living with both, one, or neither biological parent (see Table 4). None of our predictors (nor a combination thereof) improved model fit (*p* = 0.56, 0.58, 0.41, 0.65, and 0.67; see Table 6 and Fig. 5). Running these analyses with unbinned absences did not change the results (see ESM).

Post-hoc descriptive analyses indicate that outcomes among fostered and adopted children may be related to the reason the child was transferred to begin with. Children who were removed from a crisis scenario (separation, rejection, abuse, or evacuation) performed worse in exams and were absent more often (exams: mean = 56.11, SD = 25.31; absences: mean = 8.67, SD = 9.19) than children who were transferred for aspirational reasons (labor migration or education) (exams: mean = 79.45, SD = 13.39; absences: mean = 4.18, SD = 4.73; see ESM). Most children who were transferred from a crisis scenario never or rarely took part in home reading (83.3% combined), whereas most children who were transferred for aspirational reasons always or often returned their readings (85.7% combined; see ESM).

## Discussion

Child circulation has been of long-standing interest to Oceanic anthropology. Custodial alloparenting has often been presented as a puzzle for behavioral ecologists: why raise another person’s child? The subject has a produced classic paper on adoptions in Pacific Islander societies, which found the practice to be not so puzzling after all. Based on the ethnographic record, it showed that children are often transferred to kin, child circulation resolves problems associated with large families, and adoptees are disadvantaged in land inheritance (Silk, 1980). Four decades later, fostering is a resilient feature of alloparenting in rural areas of Vanuatu. Around 1 in 6 children were living away from both biological parents (17.0%), although in most cases this represents informal fostering rather than adoption. This is lower than Silk’s (1980) reported rate of adoption in Maat on Efate (31%) and resembles figures for Rotuma in Fiji (13%). However, it should be noted that our data capture a particular moment in time (how many children from a specific cohort were living away from their parents when we conducted fieldwork), whereas Silk’s (1980) figure draws on the ethnographic record and provides the proportion of individuals who had ever been adopted.

Our findings speak to the enduring importance of kinship. Extended kin (especially grandparents and aunts and uncles) are the most prominent caregivers, which is consistent with Silk’s (1980) results. Child circulation in Vanuatu is therefore an instance of kinship fostering (Isiugo-Abanihe, 1985), which supports the idea that fostering is shaped by kin altruism (Hamilton, 1963, 1964). This is not to deny that fostering is a cultural practice shaped by social norms and expectations (most importantly, moral obligations to kin). Inclusive fitness interests can shape the spread of a trait even when it is culturally transmitted; in other words, some cultural selection processes favor cultural traits because they benefit the bearer’s biological fitness (this perspective applies the theoretical framework of behavioral ecology to cultural traits without addressing processes that are not related to inclusive fitness; see Birch & Heyes, 2021; Micheletti, 2020; Micheletti et al., 2022, 2023). Our findings are also consistent with patterns seen in other Pacific Island nations, where children whose parents are unable to care for them usually live with extended kin, who act as a safety net in such situations (UNICEF, 2017:144, 153). The fact that children often lived with their grandparents aligns with the prominent role that grandparents play in childcare in many societies across the world (Aubel, 2012; Du et al., 2022; Hawkes et al., 1997; but see Page et al., 2021).

Although classic ethnographies state that residence on Efate (and in Vanuatu more broadly) is primarily patrilocal (Allen, 1981; Espirat et al., 1973), we found that most children who experienced parental absence lived with maternal kin. Most single mothers relied on support from their own kin, which aligns with qualitative findings from South Efate (Widmer, 2013). Parents who relied on custodial allomothers also showed a preference for maternal kin (the fact that this was stronger for fostered children than adoptees may come down to the small number of adoptees in our sample). This may reflect traditional norms: historically, people in northern and central Vanuatu mostly adopted children from the same descent group (Allen, 1981), so in matrilineal descent systems most adoptees probably lived with their mother’s kin. Alternatively, the rise of informal relationships without arranged marriage or bride-price (and their more frequent separation) may have weakened the responsibilities of paternal kin. Cultural preferences for maternal kin – such as matrilateral biases in alloparenting (Gaulin et al., 1997; Helle et al., 2022) – can be favored by kin altruism. This is due either to paternity uncertainty (i.e., fathers and paternal kin are less certain that children really are biologically related to them; Gaulin et al., 1997) or to the fact that support also benefits the mother, which has greater benefits for maternal kin (Perry & Daly, 2017).

This is not to say that fostering and adoption are always biased toward maternal kin. For example, Kwaio children in the Solomon Islands are usually fostered by paternal relatives, who have a right to decide these arrangements because they sponsored the father’s bride-price (Keesing, 1970). The emphasis on paternal kin appears to follow a patrilineal ideology wherein a person’s primary affiliation is with the father’s group (Keesing, 1970). Raising a brother’s sons may also strengthen the kin group, while his daughters bring in bride-price and forge marriage alliances. At the same time, both qualitative and quantitative work has shown that child-rearing practices are flexible and do not always follow the “official” norms dictated by the descent or residence system (Du & Mace, 2018; Keesing, 1970; Wentworth, 2017).

Our findings update previous work on the driving factors behind child circulation in the Pacific. Unlike Silk (1980), we did not find that child transfers serve to reduce large family sizes, which may be due to a recent decline in fertility. On Santo, we were told that in the past, many people used to have eight, ten, or more children, whereas nowadays most families only have three or four (meaning that having too many mouths to feed is less of an issue these days). Currently, child transfers are often motivated by the biological parents’ separation and/or labor migration. Most overseas labor migrants are employed as seasonal workers in the agricultural sector in Australia and New Zealand (Brickenstein, 2015). These schemes are designed to meet labor shortages, allowing workers from developing countries to take advantage of employment opportunities in stronger economies and remit money home (Brickenstein, 2015). Domestic labor migrants head to urban centers such as Port Vila. Accordingly, fostering is embedded in broader economic forces such as wealth gaps between different countries in the region, uneven development within countries, and their effects on labor markets. Additionally, the co-occurrence of separation and labor migration suggests that child circulation can fill multiple needs simultaneously. This has also been documented in Peru, where poor parents send children away to provide them with better care and education while foster parents receive support with domestic labor, which strengthens kin ties between relatives from different socioeconomic classes (Leinaweaver, 2007). Social anthropologists have long argued that adoption in Oceania is a form of “adaptive engineering” that can flexibly address many different needs and situations (Brady, 1976).

Our findings resemble trends in the Caribbean, where foster arrangements often emerge after the separation of the parents but also help families adapt to global labor markets (Nelson, 2020). As women pursue employment in other countries, children are cared for by relatives, creating transnational family networks centered on maternal kin (Nelson, 2020). Labor migration (with or without marital instability) has also been identified as an important factor in sub-Sahara Africa, Southeast Asia, and the Asia–Pacific region more broadly (Butt et al., 2017; Isiugo-Abanihe, 1985; Lloyd & Desai, 1992; Rende Taylor, 2005; Schachter & Wentworth, 2017). Along with our own, these findings illustrate that customary child-rearing practices such as adoption and fostering are not merely “traditional” but help families adapt to changing socio-ecologies: namely, socioeconomic transitions and cultural shifts, such as changing marriage practices leading to more unstable relationships. This adaptability may be the reason why child circulation is so resilient as a cultural practice. Disaggregated analyses further suggest that aspirational factors such as labor migration may be more common in informal fostering arrangements while crisis scenarios in the natal home may be more common in adoptions. This could indicate that caregivers and biological parents make strategic decisions about formalizing transfers, only transferring primary authority over a minor when the situation is dire (although because of the small number of adoptees in our sample, this is merely a tentative suggestion). Either way, custodial alloparents subsidize childcare and thus facilitate labor migration and act as a buffer when parents separate from their partners, by redistributing the costs of child-rearing within the extended family. This underscores that custodial alloparenting has important benefits for the children’s families.

At the same time, the children themselves may pay a cost. Silk (1980) found that adoptees are at risk of exclusion from land inheritance. In our sample, children whose caregivers were more invested in their education (via home reading) performed better in school, but fostered and adopted children received less support than children living with their biological parents (the best-fitting reading model only included fostering and adoption (or parental absence) as a predictor). To an extent, these results align with evidence that living away from one or both biological parents is associated with a greater risk of negative outcomes. According to Vanuatu Child Protection reports, adopted children and stepchildren are at a higher risk of sexual abuse, especially if they are female (VMJCS, 2016:13). Similar trends have been reported in Micronesia, where children living with extended kin sometimes experience abuse, domestic servitude, neglect, and exclusion from schooling (UNICEF, 2017:147; see also Berman, 2014).

But our findings also complicate this picture: no “foster effect” was found for truancy, and although fostered and adopted children’s exam scores were slightly lower, the best-fitting model did not include parental absence as a predictor. However, it did include caregiver education, and so there may be an indirect relationship as fostered and adopted children were also more likely to live with a less-educated caregiver. This may be due to generational shifts in access to schooling: grandparents often act as foster parents, but older people tend to have less formal education than the young. Ambiguous findings are not unheard of in the fostering literature: in Swaziland, fostered children are less likely to be fully vaccinated and breastfed, but their long-term nutritional status is not impacted (Sudre et al., 1990). At our field sites, children’s frequent residence with maternal kin may be protective: advice and support from maternal grandparents are often associated with improved health, socioemotional, and cognitive outcomes (Sear & Mace, 2008; Sheppard & Sear, 2016; Tanskanen & Danielsbacka, 2018; Vázquez-Vázquez et al., 2021; but see Hill & Hurtado, 2009; Madhavan & Townsend, 2007; Sear, 2008). Finally, fostering and adoption occur under a range of different circumstances that may influence outcomes (Lawson et al., 2017; Mattison et al., 2018). In our case, children who were transferred from crisis scenarios had worse educational outcomes than children who were transferred for aspirational reasons, such as labor migration. Labor migrants tend to maintain connections with their children by sending money to the foster household or visiting them periodically if they live in town. Besides, labor migration tends to be temporary, so potential gaps in investment are short-term as parents will resume caring for their children when they return, using their earnings to improve their standard of living and fund their education (Smith, 2018). In contrast, some of the adoptees in our sample have had traumatic experiences due to family conflicts related to their parents’ divorce, feelings of abandonment regarding their biological mothers, or tensions with their adoptive parents. These children often gain a reputation as “troublemakers.” This indicates that negative outcomes may not be driven by child circulation per se, but by the specific circumstances that motivate transfers. Again, the small size of our sample makes this a tentative suggestion, and at this stage our understanding of the underlying causality remains incomplete. This also applies to our understanding of the relative effects of parental absence and caregiver education because our approach selects the best model for predicting children’s educational outcomes, not for disentangling the causal mechanisms that drive these associations.

### Limitations and Future Directions

Our study has several limitations. Although fostered children are aware of their biological parentage, participants may have underreported *kastom* adoptions. We have come across some cases where guardians admitted to not informing a child about their adoption status, leading them to believe that their guardians were their biological parents. We were informed that children usually learn about their true relationship regardless, by listening in on conversations and confronting their guardians, and that this had led to conflicts in some families. Furthermore, ni-Vanuatu households can be very fluid. When families rely on seasonal labor, children may be separated from one or both of their parents for months at a time on an annual basis. As a result, the number of children who live with both biological parents continuously and without interruption is overestimated in our data set. Additionally, some of the children had transferred multiple times between different households, and because of the temporary nature of fostering, caregivers may not always view a foster child as a resident in their home. On Efate, one child was claimed by two separate households after he had recently transferred from one to the other. In a small number of interviews, the respondents initially left out a temporary resident but later included them, either prompted by our question about whether anyone else was living with them or by a reminder from the research assistants. However, since we identified the children in our sample from enrollment records and child transfers are public knowledge within the community (thanks to gossip, proximity, and interconnected kin networks in small villages), we believe that we have identified all the fostering cases among the 282 children in our study. Finally, even if neither biological parent resides in the same household as the child, they may still maintain contact. This flexibility echoes findings from the Caribbean, where child transfers “interrupt the concept of a physically bounded household consisting of unchanging members. In effect, fostered children belong to more than one household and are the responsibility of many adults” (Nelson, 2020:363).

Although we did not find that child circulation reduces large family sizes, demographic factors may still play a role in some cases. In general conversation, some informants mentioned infertility as a reason why some people adopt, and we know of childless couples who have adopted children. These adoptees are not in our sample because they were either already grown up or still infants and thus not in the age group we studied. Similarly, household size may play a role in fostered and adopted children’s outcomes, especially if they compete for strained resources with other residents in a large household. Although we have focused on rural areas, the situation may be different in towns, where smaller, nuclear, and neolocal families are becoming more common and where educated professionals make up a higher proportion of the population. Educational and demographic patterns associated with child circulation may therefore differ in cases where rural children move in with urban relatives. More broadly, children’s outcomes are best assessed by directly comparing fostered and adopted children with biological children residing in the same home, but the small size of our sample made such an analysis unfeasible. These issues should be explored in future work.

Additionally, our data narrowly focus on educational outcomes for primary schoolers. This was partly due to practical considerations since the data were collected as part of a project that focused on socio-cognitive development among children in that age group (see Brandl, 2021). Had assessments included adolescents, fostering for the purpose of attending school away from home (which was rare in our sample) probably would have been more common. Although children usually attend primary school close to their home village, there are fewer secondary schools and many of the most reputable ones are in town, meaning that adolescents must board or move in with relatives to take advantage of these opportunities (which may benefit them later in life by opening doors to white-collar work). Accordingly, we suspect that living away from both biological parents may have different reasons (and outcomes) for different age groups. Whereas infants are negatively affected by abrupt changes to breastfeeding (Wentworth, 2017), outcomes for older children may be more ambiguous (as found here), and adolescents may even benefit from the educational opportunities that fostering opens up to them. Moreover, we did not study health outcomes. In Vanuatu, health and nutrition are affected by systemic factors such as drinking water quality and infrastructure development (Morrison et al., 2020; Renzaho, 2006), over which families have little control. Although undernutrition is common in some places, outcomes vary between islands, and some are seeing growing numbers of overweight residents (Dancause et al., 2012, 2013; Morrison et al., 2020; Renzaho, 2006). This makes the selection of relevant measures more challenging, especially in studies such as ours that combine samples from more developed (Efate) and more remote (Santo) islands. Finally, outcomes for adults could reveal whether people who were adopted as children pay a long-term cost. Ethnographies suggest that adoptees are disadvantaged in land inheritance (Silk, 1980). During qualitative enquiries we found the land tenure and title system to be complex, especially on Efate (see Espirat et al., 1973). Meaningful comparisons between the landholdings of different people will require an in-depth understanding of the inheritance system. As a result, we were not able to test informants’ claims that adopted children enjoy the same land rights as biological ones.

We did not explore outcomes for the wider network of kin involved in fostering. Although natal parents benefit from easier access to wage labor and the associated cash incomes and/or can have more children with a new partner, natal siblings may gain a greater share of their parents’ resources, time, and affection. Outcomes for foster parents may depend on the reason the children were circulated in the first place. For example, foster parents may receive cash transfers and other forms of material support from biological parents who are labor migrants, as was the case in some of the families we met during our fieldwork. This is probably less common in crisis scenarios triggered by marital troubles. Benefits to foster parents may also depend on the child’s age since older children will be better able to provide meaningful support in the household. Outcomes for foster siblings may reflect household resources (with wealthier families better able to absorb an additional resident without compromising their resources) and the duration of fostering (with shorter stays less likely to have a lasting impact). Accordingly, there are probably many moving parts that influence costs and benefits for the wider network of kin, which could be explored in future work.

Future research should systematically examine how the characteristics of specific caregivers (such as age, precise kin relation, and resource availability) mediate children’s outcomes, along with the particular circumstances of transfers (crisis vs. aspirational scenarios). Such work could also tease out causality by exploring how children experience these events: in Micronesia, adoptees often experience feelings of abandonment and alienation, find transfers emotionally unsettling, and are at risk of developing mental health problems, especially when relationships are strained (Agarwal, 2017; Ierago et al., 2010; Rauchholz, 2008). Incorporating children’s perspectives will help us understand when things go right, when they go wrong, and why.

### Supplementary Information

Below is the link to the electronic supplementary material.

Supplementary file1 (PDF 211 KB)

## Data Availability

All data and code are available from the corresponding author on request.
