# Supplementary material for: Adoption, Fostering, and Parental Absence in Vanuatu
Source: Hum Nat. 2023 Aug 29;34(3):422–55. doi: 10.1007/s12110-023-09456-0 (PMC10543845; doi:10.1007/s12110-023-09456-0)
Supplement: Supplementary file 1 — Supplementary file1 (PDF 211 KB) [file 12110_2023_9456_MOESM1_ESM.pdf]

Electronic Supplementary Material for

Adoption, Fostering, and Parental Absence in Vanuatu

Eva Brandl, Emily H. Emmott, and Ruth Mace

Corresponding Author: eva_brandl@eva.mpg.de

Human Nature 34(3), 2023, https://doi.org/10.1007/s12110-023-09456-0

Contents

1. Relation between children's educational outcomes 1

2. Caregiver and household education and children's educational outcomes 2

3. Poisson models for truancy 2

4. Reason for transfer from the natal home and children's educational outcomes 2

1. Relation between children's educational outcomes

Table S1 Descriptive statistics (mean and SD) for relation between home reading participation, exam results, and truancy (0–5, 6–10, 11–20, and >20 refer to the number of absences) (children with known exam scores and absences: n = 176; children with known reading participation: n = 91)

	**Truancy**	**Exams**
**Truancy**		
0-5	—	75.94 (16.15)
6-10	—	71.09 (18.47)
11-20	—	66.06 (21.75)
>20	—	51.75 (36.67)
**Readings**		
Never	11.75 (8.66)	46.08 (17.18)
Rarely	9.71 (8.83)	57.71 (22.15)
Sometimes	3.53 (2.33)	67.27 (17.47)
Often	3.27 (3.14)	66.10 (18.71)
Always	3.19 (3.01)	77.63 (12.26)

2. Caregiver and household education and children's educational outcomes

Table S2 Comparison of exam scores and truancy (mean, SD), and home reading participation (% of children) by caregiver- and household-level education (Primary = up to 6 years of education, Lower Sec. = 7–10 years of education, Upper Sec. = 11 years of education or more; children with known exam scores and absences: n = 176; children with known reading participation: n = 91)

	**Caregiver Education**			**Household Education**		
	**Primary**	**Lower Sec.**	**Upper Sec.**	**Primary**	**Lower Sec.**	**Upper Sec.**
**Exams**	68.63 (20.26)	74.38 (16.53)	77.54 (17.79)	72.28 (21.19)	71.52 (16.53)	73.86 (19.44)
**Truancy**	6.09 (8.07)	5.70 (5.19)	4.27 (3.49)	7.20 (11.07)	4.80 (4.42)	5.65 (5.49)
**Readings**						
Never	18.9%	11.8%	5.0%	10.0%	13.9%	13.3%
Rarely	10.8%	5.9%	5.0%	10.0%	8.3%	6.7%
Sometimes	16.2%	20.6%	10.0%	30.0%	19.4%	11.1%
Often	24.3%	41.2%	35.0%	10.0%	38.9%	33.3%
Always	29.7%	20.6%	45.0%	40.0%	19.4%	35.6%

3. Poisson models for truancy

Table S3 Model comparison: truancy predicted by caregiver- and household-level education and the number of biological parents in the home (children with known absences, n = 176), unbinned outcome data

**Models**	**df**	**AIC**	**Log-Like.**	χ^2	p
**Truancy**					
Baseline	—	979.04	−486.52	—	—
Caregiver education	2	981.20	−485.60	1.84	.40
Caregiver and household education	4	981.98	−483.99	5.06	.28
Parental absence	1	980.57	−486.29	0.47	.49
Parental absence and caregiver education	3	982.92	−485.46	2.12	.55
Parental absence and c. and h. education	5	983.81	−483.91	5.23	.39

4. Reason for transfer from the natal home and children's educational outcomes

Table S4 Comparison of exam scores and truancy (mean, SD) and home reading participation (% of children) between children transferred for aspirational reasons (labor migration or education), children transferred for crisis reasons (separation, rejection, abuse, or evacuation), and children transferred for unknown or mixed reasons (labor migration combined with separation or no reason provided). Figures only include fostered and adopted children with known exam scores and truancy (n = 34) or home reading participation rates (n = 20)

	**Reason for Transfer**		
	**Crisis**	**Aspirational**	**Mixed or Unknown**
**Exams**	56.11 (25.31)	79.45 (13.39)	66.43 (21.26)
**Truancy**	8.67 (9.19)	4.18 (4.73)	5.07 (4.73)
**Readings**			
Never	50.0%	0.0%	28.6%
Rarely	33.3%	14.3%	0.0%
Sometimes	0.0%	0.0%	0.0%
Often	16.7%	71.4%	71.4%
Always	0.0%	14.3%	0.0%
